# Benefits of Taurisolo in Diabetic Patients with Peripheral Artery Disease

**DOI:** 10.3390/jcdd11060174

**Published:** 2024-06-04

**Authors:** Bruno Amato, Ettore Novellino, Davide Morlando, Camilla Vanoli, Emilio Vanoli, Fulvio Ferrara, Rossana Difruscolo, Vito Maria Goffredo, Rita Compagna, Gian Carlo Tenore, Mariano Stornaiuolo, Mario Fordellone, Eugenio Caradonna

**Affiliations:** 1 Department of Public Health, Università degli Studi di Napoli Federico II, 80138 Naples, Italy; bruno.amato@unina.it (B.A.); morlandodavide@gmail.com (D.M.); 2 Chimica Farmaceutica e Tossicologica, Università Cattolica del Sacro Cuore, 20123 Rome, Italy; ettore.novellino@unicatt.it; 3 Clinical Psychology, Antioch University Los Angeles, Culver City, CA 90230, USA; 4 School of Nursing, University of Pavia, 27100 Pavia, Italy; emivano@gmail.com; 5 Centro Diagnostico Italiano, Department of Clinical Laboratory, 20100 Milan, Italy; fulvio.ferrara@cdi.it (F.F.); eugenio.caradonna@cdi.it (E.C.); 6 Biotecnologie Mediche e Farmaceutiche, Università degli Studi di Bari, 70126 Bari, Italy; rossanadifruscolo@gmail.com; 7 Department of Interdisciplinary Medicine, Università degli Studi di Bari, 70124 Bari, Italy; vitogoffredo93@gmail.com; 8 Vascular Surgery Unit AORN Ospedale dei Colli, 80131 Naples, Italy; rita.compagna@gmail.com; 9 Department of Pharmacy, Università degli Studi di Napoli Federico II, 80138 Naples, Italy; gctenore@unina.it (G.C.T.); mariano.stornaiuolo@unina.it (M.S.); 10 Unità di Statistica Medica, Dipartimento di Salute Mentale e Fisica e Medicina Preventiva, Università degli Studi della Campania ‘Luigi Vanvitelli’, 81020 Napoli, Italy; mario.fordellone@unicampania.it

**Keywords:** Taurisolo^®^, trimethyl-*N*-oxide (TMAO), peripheral artery disease (PAD), claudication, diabetes

## Abstract

Trimethyl-*N*-oxide (TMAO) has been linked to peripheral artery disease (PAD). Taurisolo^Ⓡ^ is a natural, balanced phytocomplex containing resveratrol, quercetin, catechins, procianidins, gallic acid, and caffeic acid. Numerous studies have shown that Taurisolo^Ⓡ^ reduces the damage of TMAO and exerts a protective effect on endothelial cells (ECs). The aim of this randomized, double-blind, single-center study was to evaluate the effects of Taurisolo^Ⓡ^ on claudication in patients with PAD (Rutheford grade I, category II, Fontaine Classification: Stage IIA, American Medical Association Whole Person Impairment Classification: Class 0—WPI 0%) in two parallel groups of 31 patients. The primary outcomes were an increase in the pain-free walking distance and the ankle/brachial pressure index at the beginning and at the end of the treatment with Taurisolo. The secondary endpoint was the serum TMAO changes. The claudication distance improved by 14.1% in the Taurisolo group and by 2.0% in the placebo group, while the maximal distance increased by 15.8% and 0.6% only, respectively (both *p* < 0.05). The TMAO plasma levels decreased from 3.97 ± 2.13 micromole/L to 0.87 ± 0.48 (*p* < 0.0001) in the treated group. All these changes were highly significant both in univariate mixed models as well as in the adjusted model. Ultimately, Taurisolo^Ⓡ^ might be an effective intervention to ameliorate intermittent claudication.

## 1. Introduction

Peripheral artery disease (PAD) is a global pandemic disease and represents a growing health matter [1,2]. A substantial number of subjects with asymptomatic PAD show progression and develop intermittent claudication within a year of its first manifestation [3]. Furthermore, patients with symptomatic PAD have a 2.5 higher mortality risk over the 5 years after onset when compared to a reference population [4]. The clinical pictures of PAD range from asymptomatic disease to the appearance of ischemic muscle pain on exertion (intermittent claudication, IC) up to critical ischemia of the lower limbs (critical limb ischemia, CLl) with the presence of pain at rest, necrosis, and gangrene. The annual incidence of CLI has a wide spectrum, ranging from 220 to 3500 cases/1,000,000 inhabitants. Diabetes is a risk factor for peripheral artery disease (PAD), and when present, is associated with poorer outcomes [5]. The presence of PAD in diabetic patients is associated with a higher risk of developing foot ulcers [6].

The main objectives in the integrated management of mild claudication are the prevention of major cardiovascular events and the slowing of disease progression. In patients with PAD and symptoms of IC, the intake of antiplatelet drugs (the most frequently used is acetylsalicylic acid (ASA)) and Cilostazol is the standard strategy to reduce pain and improve function and quality of life [7]. In addition to antiplatelet therapy, polyphenols such as quercetin, anthocyanins, and resveratrol appear effective mainly because of their antioxidant action [8,9,10,11], thus reducing oxidative stress (OxS). Moreover, polyphenols exert a protective action on endothelial cells and on circulating angiogenic cells [12,13].

The multiple beneficial effects of polyphenols triggered the interest to develop a supplement with dual efficiency: adequate concentration of polyphenols and absence of side effects. “Taurisolo’’ was the name attributed to a phytocomplex of polyphenols extracted from the pomace of Aglianico grapes. Taurisolo^Ⓡ^, an agri-food by-product rich in polyphenols, has a balanced composition of resveratrol, quercetin, catechins, procianidins, gallic acid, and caffeic acid [14,15].

The target of Taurisolo^Ⓡ^ is trimethylamine (TMA), a molecule produced by the gut microbiota from choline, carnitine, lecithin, gamma-butyrobetaine, and phosphatidylcholine. TMA originates from foods abundant in choline, L-carnitine, and phosphatidylcholine [16] such as red meat, saltwater fish, eggs, and dairy products and partly from fruits, vegetables, and grains [17], and it is converted to trimethylamine-*N*-oxide (TMAO) through specific bacterial enzymes. The known enzyme pathways are as follows: choline TMAO lyase, (cutC/D), carnitine monooxygenase (cntA/B), betaine reductase, and TMAO reductase [18,19]. Trimethylamine is mostly oxidized to TMAO in the liver by flavin monooxygenase 3 (FMO3), which is five times more active than flavin monooxygenase 1 (FMO1), which is expressed primarily in the kidneys and intestines [20,21,22].

A key effect of TMAO is to facilitate the onset of atherosclerosis through several complex mechanisms, and it is a known marker of arteriosclerotic vascular disease [23].

TMAO activates the cytosolic inflammasome NOD-Like Receptor Protein 3 (NLRP3), one of the key processes in the genesis of atherosclerosis and many chronic diseases [24], and accelerates the senescence of endothelial cells by inhibiting the activity of sirtuin 1 (SIRT1) [25,26,27].

Also, TMAO increases the expression of scavenger receptors (CD 4 and SR-A1) in macrophages, with a consequent increase in cholesterol due to a reduction in reverse transport as well as the number of “foam cells” in arteriosclerotic lesions, with an increase in the thickness of the tunica media of the carotid artery [28,29,30].

Plasma TMAO predicts the risk of adverse events in patients with peripheral vascular disease [31] and inhibits angiogenesis and perfusion in patients with PAD [32].

TMAO levels are abnormally high in diabetic patients as well as during continuous statins treatment, which may contribute to the development and progression of atherosclerosis [33], and Taurisolo^Ⓡ^ might reduce TMAO serum levels [34]. The progression of peripheral artery disease is more rapid in diabetic patients.

Polyphenols could improve the endothelial dysfunction present in diabetic endothelial cells [35,36].

This background prompts the present study, aimed at testing whether Taurisolo^®^ can reduce the progression of peripheral arterial disease of the lower limbs (PAD) in diabetic patients with Rutheford grade I, category II (Fontaine Classification: Stage IIA, American Medical Association Whole Person Impairment Classification: Class 0—WPI 0%), who are frequently poor or non-responders to the currently available therapy.

## 2. Materials and Methods

This was a single-center, randomized, placebo-controlled, parallel-group, follow-up, double-blind study and was approved by the Ethic Committee of the University of Naples Federico II, Naples, Italy.

Taurisolo^Ⓡ^, was administered orally (400 mg b.i.d.) as a supplement to standard therapy with ASA and Cilostazol.

The primary aim was to test the effects of Taurisolo^®^ on pain-free walking (painful muscular symptoms linked to muscular effort) using the “six-minute walking test (6MWT)” (t0, t1, t2, t3, and t4), with annotation of the following parameters (Table 1):

- Initial claudication distance (ICD);
- Distance that requires walking to stop (ACD, absolute claudication distance);
- Recovery time (RT): rest time needed to resume walking.

The primary outcome was remotely evaluated using a freely downloadable Pedometer app to measure the following:

- The initial claudication distance (ICD);
- The distance reached before stop walking (ACD, absolute claudication distance);
- Recovery time (time needed to resume exercise).

The secondary objectives focused on the changes from TMAO’s reduction on the metabolic parameters, lipid structure, and renal function.

In addition to the TMAO levels, the following information was collected (Table 1):

- Blood sugar, HbA1c, blood count, QPE, ESR, total cholesterol, HDL, and LDL;
- Renal function and coagulation structure;
- The ABI (ankle/brachial index) pressure index;
- Quality of life by the administration of the SF-36 “Short-Form 36 items health Survey” questionnaire;
- Evaluation of the duration of the therapeutic effects, if any, of “increased walking ability”, three months after the suspension of therapy with Taurisolo^®^ (t4).

The experimental groups were as follows:

Taurisolo group: Taurisolo^®^-based food supplement (400 mg b.i.d);

Placebo group: subjects who took two gastro-resistant placebo tablets/day, each containing 400 mg of maltodextrin.

Standard therapy consisted of the following:

- Acetylsalicylic acid (ASA), 100 mg, per day (after lunch);
- Cilostazol, 50 mg orally daily (after breakfast).

The inclusion criteria were as follows:

- Diabetes, on insulin therapy and/or oral antidiabetics;
- Presence of intermittent claudication of a lower limb of vascular origin with a walking perimeter greater than 200 m (Rutheford grade I, category II for intermittent claudication), with absence of indication for revascularization;
- Positive Doppler ultrasound examination for steno-obstructive lesion(s) of the limb affected by the claudication;
- Age range from 40 to 80 years old;
- Possession of a device compatible with the Pedometer app;
- Consent to install the Pedometer app on a personal device;
- Ability and willingness to comply with all protocol requirements, including the use of the Pedometer app;
- Participating in therapy with ASA and Cilostazol;
- ABI of the affected limb < 0.7;
- Ability to move independently.

The exclusion criteria were as follows:

- Joint pathologies that impaired or prevented walking;
- Age over 80 years;
- Under nutraceutical treatment based on pomace polyphenols in the three months prior to recruitment (as they could create a synergism with the study treatment);
- Women who were pregnant, suspect pregnancy, or planning pregnancy;
- Women breastfeeding;
- Disabling heart disease and heart failure;
- Symptomatic chronic obstructive pulmonary disease;
- Muscle diseases;
- Demyelinating diseases;
- Current chronic infectious diseases;
- Previous vascular surgeries;
- Allergies, hypersensitivity, or contraindications to one or more components present in the food supplement;
- Current oncological status and under treatment;
- Included in the patient selection criteria was the exclusion of those with spinal stenosis, based on the consistency of concomitant clinical characteristics of claudication and the ABI.

The enrolled subjects were given the information sheet, with clear information regarding the clinical study, the objectives, and methods of implementation. During the screening visit (Vs), several pieces of information were collected (Table 1) The follow-up was scheduled at t0 (one week after the screening visit), t1 (one month of treatment), t2 (three months of treatment), t3 (six months of treatment), and at t4 (three months after discontinuation of treatment) (Table 1).

Physical activity was defined as follows: walking until the onset of symptoms (greater than or equal to 200 m), 5–10 times per day, with a frequency of 5 times per week. Physical activity was monitored using a Pedometer application.

The reported smoking prevalence in the patient sample was indicative of an addiction that posed a significant challenge to overcome. Despite encouragement for patients to either stop or reduce their frequency smoking, no cessation or reduction was observed during the observation period, as the reliability of patient responses was deemed questionable.

The randomization was performed by a statistician using STATA 16 software (Stata Statistical Software: Release 16. StataCorp LLC, College Station, TX, USA).

### 2.1. Determination of Circulating Levels of TMAO

The serum samples were stored at −80 °C. Upon defrosting, serum proteins were precipitated with two volumes of cold methanol and centrifuged at 14,000× *g* for 10 min (4 °C). The supernatants were collected and analyzed using high-performance liquid chromatography–mass spectrometry (HPLC-MS) with a Jasco Extrema LC-4000 system (Jasco Inc., Easton, MD, USA) coupled to a single quadrupole mass spectrometer (Advion ExpressIonL CMS, Advion Inc., Ithaca, NY, USA), which was equipped with an electrospray ionization (ESI) source operating in positive ion mode. The chromatographic separation was performed using a Luna HILIC column (150 × 3 mm, 5 µm particles) in combination with a guard column (HILIC), both supplied by Phenomenex (Torrance, CA, USA).

### 2.2. Statistical Analysis and Sample Size 

Continuous variables were reported as either means and the standard deviation or median and inter-quartile ranges (IQRs) according to their distribution, as assessed by the Shapiro–Wilk normality test. Categorical variables were reported as absolute frequencies and percentages. Differences in the baseline characteristics between the treated group and control group were tested using the Student’s *t*-test or the Kruskal–Wallis test (according to their distribution) for continuous variables, and Pearson chi-squared or Fisher’s exact tests for categorical variables. 

Two linear mixed models (i.e., one unadjusted univariate model and one that adjusted for clinical variables (multivariate model)) for each endpoint (i.e., ICDm, ACDm, and TMAO) were performed to estimate the covariate effects. The covariates included in the model were the treatment groups (i.e., Taurisolo vs. placebo) and time points (i.e., from baseline to T4) as the categorical variables and their interactions for the unadjusted models. Subsequently, these model estimates were adjusted for clinical variables (i.e., gender, age, BMI, glycemia, total cholesterol, azotemia, and creatinine). The selection of clinical variables was suggested by the BIC and AIC evaluation criteria.

Statistical tests with *p*-values smaller than 0.05 were considered statistically significant. All the statistical analyses were performed with R Studio Statistical software, version 4.1.3.

## 3. Results

The total duration of the study was 12 months, specifically 3 months for patient enrollment and 6 months of treatment with a 3-month follow-up. The study began in January 2022 with the enrollment phase, continued with the treatment phase at t0 after approval from the Ethics Committee of the Federico II University on 16 March 2022 (Protocol No.: 12008 of 16 March 2022), and ended in January 2023. 

A total of 75 subjects were screened for eligibility. Of these, 13 (17.33%) were excluded. The selected 62 patients were randomized to receive Taurisolo^®^ (n = 31) or the placebo (n = 31). Revascularization was deemed not indicated for this group of patients (Rutheford class I–II) by our vascular team according to the current guidelines.

Eleven patients (17.7%, five in Taurisolo group and six in Placebo group) withdrew for unwillingness to adhere to the protocol. Overall, 26 patients in each group completed the study (Table 2 and Table 3). Concomitant diseases were coronary artery disease, diabetes, renal impairment, hypertension, and chronic obstructive pulmonary disease.

The localization of atherosclerosis in the peripheral circulation was evaluated using Doppler ultrasound examination of the lower limbs (obstructive lesions mainly of sector B-steno–femoral obstructions, 76%, and A + B mixed iliac–femoral stenosis, 24%). 

**Figure 1:**
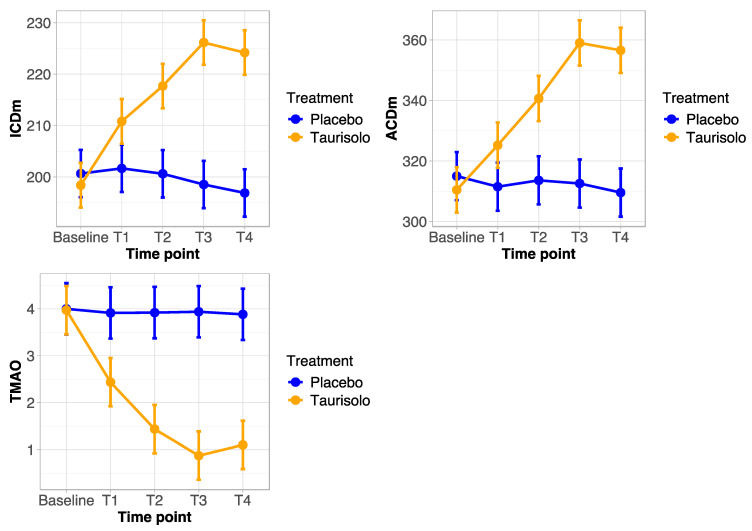
Endpoint trends from baseline to T4 time point for the treated (Taurisolo) and control (placebo) groups. (**Top left**): ICDm endpoint; (**top right**): ACDm endpoint; (**bottom left**): TMAO endpoint.

Taurisolo^Ⓡ^ met the primary goal of the study, as both ICD and ACD increased by 14.1 and 15.8%, respectively (*p* < 0.05) at t3 (6 months of treatment) compared to the screening visit values (Table 4). The ICD values increased from 198 ± 23 m to 226 ± 23 m at t3, (+14.1% compared to the placebo +1.0%), while after 9 months (i.e., after 3 months of follow-up, at t4) there was an increase of +13.1% (from 198 ± 23 m to 224 ± 23 m) compared to the beginning of the study (Figure 1, Table 4).

At the same time of the progressive improvement of the 6 MWT, the serum TMAO levels decreased with Taurisolo^®^, while no changes occurred in the placebo group (Table 5). Of note, the effects of Taurisolo^®^ remained significant even at t4, namely at 6 months after treatment was stopped (Table 4 and Table 5). Pearson’s test documented a strong correlation between the TMAO plasma level decrease induced by Taurisolo^®^ and the amelioration of symptoms and walking performance in the treated population.

The quality of life (QoL) also increased with Taurisolo^®^. Specifically, the domains that evaluated the physical condition (AF, DF, SG, and VT) and the domains that evaluated the mental condition (RE, AS, VT, and SM) progressively improved with active therapy. Arterial flow with the ankle/brachial pressure index (ABI) did not change (+2.95% between t0 and t3).

In Figure 1, the endpoint trends for the two groups of patients are shown. In particular, in the top-left panel, the ICDm trend from the baseline time point to t4 was shown, in top-right panel, there was an ACDm trend, and in bottom-left, there was a TMAO trend. In these line plots, the points represent the means of the groups, while the bars represent the confidence intervals at a 95% confidence degree. Figure 1 shows the high effect of the Taurisolo treatment by time point for each endpoint. In particular, we can see a positive impact for ICDm and ACDm, whereas a negative impact on TMAO is shown.

The results obtained using the univariate linear mixed model on the ICDm response variable showed a statistically significant positive effect of the interaction between the Taurisolo treatment and time point (*p*-value < 0.0001). The same results were obtained with the linear mixed model adjusted for the selected clinical variables (i.e., *p*-value < 0.0001). Similar results were obtained using the univariate linear mixed model estimates on the ACDm response variable, where a high significance of the interaction term (*p*-value < 0.0001) was shown. The results were also confirmed in the adjusted model (*p*-value < 0.0001). Finally, the univariate linear mixed model estimates on the TMAO response variable showed a statistically significant negative effect of the interaction between Taurisolo treatment and the time point (*p*-value < 0.0001). The adjusted model showed the same results (*p*-value < 0.0001).

## 4. Discussion

Peripheral arterial disease (PAD) and diabetes pose significant challenges to the healthcare system, and their co-occurrence typically results in a poor prognosis, which is exacerbated by the rapid progression of PAD in individuals with diabetes [5,37,38]. In this context, the present study provides novel evidence indicating that a supplementary phytocomplex may decrease the TMAO plasma level and significantly affect the walking performance and quality of life of patients with diabetes and PAD.

Recent laboratory research and clinical data have highlighted the connection between microbiota and arteriosclerosis [39].

TMA has gained particular attention, being produced by Firmicutes and Proteobacteria, meta-organisms of quaternary amines present in food as choline, carnitine, and phosphatidylcholine [40].

Adherence to diet is an important aspect of therapy for patients with diabetes and arteriosclerosis [41].

However, it is important to note that adherence to dietary recommendations can be challenging for patients with chronic conditions such as arteriosclerosis [42,43].

In normal circumstances, TMAO plays a crucial role in regulating fluid balance, stabilizing protective functions and aiding in preserving the integrity of cells [44]. However, TMAO has been identified as an inflammatory and proatherogenic molecule when present at a value over the physiologic range and impairs angiogenesis, repressing the CXCR4 axis [45,46,47,48,49].

TMAO has been demonstrated to augment foam cell formation by stimulating the uptake of cholesterol and low-density lipoprotein (LDL) by macrophages.

Moreover, TMAO impairs cholesterol efflux mechanisms, preventing the removal of cholesterol from macrophages and promoting their transformation into foam cells [50]. TMAO induces platelet activation and hyperactivity and can reduce the efficacy of clopidogrel [51,52].

In our study population, the TMAO levels at enrollment were 3.97 ± 2.13 in the Taurisolo group and 3.86 ± 1.82 in the controlled group. Elevated TMAO values are associated with an increased incidence of major cardiovascular events over 5 years follow-up. A reduction in TMAO in the physiologic range could result in the slower progression of the disease and in the improvement of the clinical status.

This placebo-controlled study proved that Taurisolo^®^ significantly enhances the pain-free walking distance in individuals diagnosed with intermittent claudication caused by peripheral artery disease. Following a 6-month treatment, the initial claudication distance (ICD) increased by 14.1% and the absolute claudication distance (ACD) increased by 15.8%, as opposed to minimal changes in the placebo group. Moreover, treatment with Taurisolo^®^ resulted in substantial reductions in the serum levels of TMAO, while TMAO levels remained unchanged in the placebo group. Pearson’s correlation analysis documented a linear relationship between TMAO—ICD and TMAO—ACD at times t0, t3, and t4, having obtained an always lower “r” coefficient to −0.9.

Two mechanisms of action by which Taurisolo^®^ may exert its TMAO-reducing effect could be through antioxidant activity and microbiota remodeling, both exerted by polyphenols [53,54,55].

Taurisolo^®^ led to improvements in lower extremity muscular endurance in patients with claudication, while the ankle/brachial index had no significant changes. This apparent incongruence might reflect a specific action on muscular performance by Taurisolo^®^ that is related to its antioxidant effects, while the benefits on endothelial function may become significant at a later time. As a matter of fact, prior research has shown poor correlation between ABI changes and clinical improvements from medical therapy in claudication [56]. Therefore, the functional assessments of pain-free walking provide the most meaningful evidence of the beneficial effects of Taurisolo^®^.

It is also worth highlighting the enhancement in the level of QoL, as detected by the SF 36 questionnaire and in the parameters (domains) concerning the sphere of mental and physical health.

Overall, the benefits observed are probably due to the properties of the specific polyphenols contained in Taurisolo^®^, such as its antioxidant and anti-inflammatory effects, which have been shown to enhance endothelial function, inhibit platelet aggregation, and reduce atherosclerotic plaque formation [13,57,58,59], combined with its direct effect on TMAO plasma levels. Indeed, reducing this proatherogenic metabolite has certainly contributed to the benefits seen in this study.

It is noteworthy that the improvements seen in the pain-free walking distance persisted for 3 months after discontinuing Taurisolo^®^ treatment. This suggests a sustained benefit rather than just an acute effect while partaking in treatment. One possible explanation is a lasting reduction in oxidative stress, inflammation, or plaque burden that continues to enhance peripheral perfusion. One other relevant aspect could be the reshaping of the microbiota from polyphenols [35,36,54]. Further imaging or biomarker studies would be necessary to investigate the durability of these benefits after the cessation of Taurisolo^®^.

## 5. Conclusions

This study was conducted at a single center but provides convincing evidence that Taurisolo^®^ can increase pain-free walking in patients with claudication while significantly reducing risk factors such as TMAO.

Further studies of a longer observation duration, and possibly accompanied by vascular imaging tests, would be appropriate to better understand the mechanisms by which a phytocomplex of polyphenols acts in reducing TMAO and whether this reduction, in addition to blocking the progression of atherosclerosis, can also determine a regression of atherosclerosis and its clinical manifestations in patients with PAD and specifically in those with diabetes.

## Figures and Tables

**Table 1:** Study Schedule.

	Screening (Vs)	V1 (t0)	V2 1-Month Treatment (t1)	V3 3-Month Treatment (t2)	V4 6-Month Treatment (t3)	V5 3-Month Follow-Up (t4)
Informed Consent	X					
Demographics	X					
Eligibility (inclusion/exclusion criteria): - General clinical details and *anthropometric evaluation* * - Anamnesis and objective examination - Characteristics of claudicatio intermittens - Echo-doppler of lower limbs and ABI	X					
Blood sample to evaluate the following: - Serum levels of TMAO - *Metabolic* * and *renal* * function parameters - SPEP, ESR, blood count, and *coagulation tests* *	X	=Vs	X	X	X	X
Short Form-36 health survey		X	X	X	X	X
ABI (Ankle/brachial index)	X	=Vs	X	X	X	X
6MWT (Six-minute walking test)	X	=Vs	X	X	X	X
Dispensing food supplement (grape pomace polyphenols) or placebo		X		X		
Appearance of disorders and/or adverse events			X	X	X	X
Compliance with treatment			X	X	X	X
Monitoring interactions with drugs in use			X	X	X	X
Monitoring major vascular complications			X	X	X	X

*Anthropometric evaluation* *: weight (Kg), height (m), waist circumference (cm), and hip circumference (cm). *Metabolic parameters* *: glycemia, glycated hemoglobin (HbA1c), total cholesterol, HDL and LDL, and triglycerides. *Renal function parameters* *: azotemia, creatinine, and electrolytes. *Coagulation tests* *: PT and PTT.

**Table 2:** Baseline characteristics of study participants.

Characteristics	Taurisolo (n = 26)	Placebo (n = 25)	*p*-Value
Male gender n = 21 (41%)	n = 11	n = 10	
Female gender n = 30 (59%)	n = 15	n = 15	
Age (year)	59.6 ± 6.5	60.6 ± 7.4	0.6101
Weight (Kg)	75.6 ± 9.8	78.3 ± 11.8	0.3776
Height (m)	1.65 ± 0.08	1.67 ± 0.08	0.3765
BMI (kg/m^2^)	27.7 ± 2.5	27.9 ± 2.9	0.7928
WC (cm)	100 ± 13	103 ± 20	0.5267
HC (cm)	106 ± 7	108 ± 13	0.4948
WHR (cm)	0.94 ± 0.09	0.95 ± 0.10	0.7088

Statistical significance was calculated with Student’s *t*-test, with α = 0,05 and *p*-value < 0,05.

**Table 3:** Main blood tests during screening visit (SV) and at t3 in groups 1 and 2.

	1. Taurisolo Group			2. Placebo Group		
Blood Tests	SV	t3	*p* Value	SV	t3	*p* Value
RBC mil/mm^3^	4.394 ± 0.25	4.381 ± 0.27	0.8578	4.339 ± 0.23	4.352 ± 0.24	0.8458
WBC thou/mm^3^	7.641 ± 1.8	7.595 ± 1.7	0.9249	7.797 ± 1.6	7.685 ± 1.5	0.7996
Platelets thou/mm^3^	257 ± 83	249 ± 69	0.7071	241 ± 93	245 ± 87	0.8759
HGB gr/dL	13.4 ± 0.6	13.1 ± 0.5	0.0558	13.3 ± 0.5	13.4 ± 0.6	0.5251
Glycemia mg/dL	97.5 ± 13.8	96.9 ± 13.5	0.8747	95.4 ± 10.7	96.8 ± 11.8	0.6623
HbA1c %	9.1 ± 1.6%	9.3 ± 1.7%	0.6641	8.3 ± 1.5%	8.6 ± 1.6%	0.4973
Total cholesterol mg/dL	198 ± 13	192 ± 14	0.1156	197 ± 16	196 ± 15	0.8206
Cholesterol HDL mg/dL	44 ± 8	48 ± 7	0.0607	46 ± 6	47 ± 5	0.5251
Cholesterol LDL mg/dL	131 ± 14	126 ± 13	0.1881	129 ± 17	128 ± 14	0.8214
Triglycerides mg/dL	117 ± 16	113 ± 11	0.2986	110 ± 17	109 ± 15	0.8264
Azotemia mg/dL	41 ± 8	42 ± 7	0.6335	44 ± 8	43 ± 6	0.6194
Creatinine mg/dL	0.87 ± 0.23	0.88 ± 0.27	0.8863	0.88 ± 0.19	0.89 ± 0.23	0.8676
Na mmol/L	140 ± 2	139 ± 3	0.1635	139 ± 2	140 ± 3	0.1719
K mmol/L	4.4 ± 0.6	4.3 ± 0.5	0.5168	4.3 ± 0.5	4.1 ± 0.4	0.1249
ESR mm/h	8.5 ± 2.9	8.8 ± 2.6	0.6962	8.3 ± 3.4	8.6 ± 2.8	0.7349
Albumin g/dL	4.2 ± 0.2	4.1 ± 0.3	0.1635	4.1 ± 0.2	4.0 ± 0.3	0.1719
PT %	88.9 ± 9.6%	90.3 ± 10.1%	0.6107	88.1 ± 11.9%	88.9 ± 11.3%	0.8085
PTT sec.	62.7 ± 4.4	62.9 ± 4.1	0.8660	63.9 ± 5.1	62.9 ± 4.4	0.4615

Statistical significance was calculated with Student’s *t*-test, with α = 0.05 and *p*-value < 0.05.

**Table 4:** ICD and ACD during follow-up.

	t0	t1	t2	t3	t4	Δ% (t0–t3)	*p*-Value (t0–t3)
ICD (m)							
1. Taurisolo group	198 ± 23	211 ± 24	218 ± 24	226 ± 23	224 ± 23	~14.1%	0.0001
2. Placebo group	201 ± 26	203 ± 26	204 ± 26	205 ± 25	203 ± 26	~2.0%	0.5818
ACD (m)							
1. Taurisolo group	310 ± 30	325 ± 30	341 ± 31	359 ± 32	357 ± 32	~15.8%	0.0001
2. Placebo group	315 ± 24	316 ± 28	318 ± 28	319 ± 27	317 ± 28	~0.6%	0.5824

Statistical significance was calculated with Student’s *t*-test, with α = 0.05 and *p*-value < 0.05.

**Table 5:** TMAO serum levels over the 4 study visits.

	Taurisolo µmol/L	Placebo µmol/L
TMAO a vs. = t0	3.97 ± 2. 13	3.86 ± 1.82
TMAO at t1	2.44 ± 1.14	3.8 ± 1.71
TMAO ta t2	1.44 ± 0.64	3.76 ± 1.55
TMAO at t3	0.87 ± 0.48	3.85 ± 1.66
TMAO at t4	1.1 ± 0.54	3.78 ± 1.48
Δ % (t0–t3)	−78%	−0.26%
*p* (t0–t3)	0.0001	0.9848
Δ % (t0–t4)	−72%	−2.01%
*p* (t0–t4)	0.0001	0.8653

## Data Availability

Data are contained within the article.
